# Heart rate variability findings in neurological decompression sickness: an exploratory case series

**DOI:** 10.3389/fphys.2026.1826066

**Published:** 2026-06-10

**Authors:** Gerald Schmitz

**Affiliations:** Centro de Medicina Hiperbárica OHB, Hyperbaric Medicine Service, Hospital CIMA, San José, Costa Rica

**Keywords:** autonomic nervous system, decompression sickness, heart rate variability, hyperbaric oxygen therapy, nonlinear dynamics

## Abstract

**Background:**

Neurological decompression sickness (NDCS) is a potentially severe complication of diving, but its effects on autonomic cardiovascular regulation remain poorly understood. Heart rate variability (HRV) has emerged as a noninvasive method for assessing autonomic nervous system dynamics in both physiological and pathological conditions.

**Objective:**

To describe multidomain HRV patterns in divers presenting with NDCS prior to hyperbaric oxygen therapy (HBOT).

**Methods:**

This exploratory case series included eight divers diagnosed with NDCS at a tertiary hyperbaric medicine center. Continuous electrocardiographic monitoring was performed before HBOT, and HRV analysis was conducted using a standardized offline processing pipeline. Time-domain (SDNN, RMSSD, pNN50), frequency-domain (HF power, LF/HF ratio), and nonlinear metrics (Shannon entropy and two-dimensional Poincaré analysis) were evaluated. HRV parameters were analyzed both as absolute values and as Z-score normalized values relative to published reference populations.

**Results:**

Conventional HRV indices demonstrated marked interindividual variability across cases. Some patients exhibited elevated global variability and vagally associated indices, whereas others showed reduced or near-reference values. Entropy-based measures also varied across the cohort, with near-reference values in some cases and lower values in others, particularly Cases 7 and 8. These findings suggest heterogeneous alterations in cardiac rhythm dynamics rather than a uniform HRV phenotype.

**Conclusions:**

NDCS may be associated with measurable but heterogeneous disturbances in autonomic cardiovascular regulation detectable through HRV analysis. Entropy-based measures may provide exploratory information on NN interval distributional irregularity, but these findings should be interpreted cautiously because of the small sample size, retrospective design, spontaneous respiration, and methodological sensitivity of entropy estimates. Larger prospective studies are needed to determine whether HRV can contribute to physiological characterization or monitoring of decompression illness.

## Introduction

Heart rate variability (HRV) is a widely used noninvasive marker of autonomic nervous system regulation in both clinical medicine and physiology research. Across multiple medical fields, HRV has been proposed as a useful tool for physiological monitoring, prognostic assessment, and early detection of disease (Addleman et al., 2025). In acute care settings, reductions in HRV are consistently associated with illness severity and mortality, and short-term HRV measurements have been shown to predict outcomes in conditions such as sepsis and critical illness (Karmali et al., 2017; de Castilho et al., 2018; Liu et al., 2021; Keng et al., 2025; Huang et al., 2025). Continuous HRV monitoring may also precede clinical deterioration by several hours to days (Ahmad et al., 2009; Bodénes et al., 2022).

In diving physiology, immersion and increased ambient pressure induce characteristic autonomic responses, typically including bradycardia and increased parasympathetic modulation related to activation of the diving reflex (Schipke et al., 2001; Lafère et al., 2021; Vulić et al., 2024; Noh et al., 2018). However, environmental stressors, task load, and repeated dives can produce more complex patterns of autonomic activation (Flouris and Scott, 2009; Schaller et al., 2021; Berry et al., 2017). Additional studies and reviews have evaluated HRV during underwater exercise, cold-water diving, simulated diving, and recreational diving using continuous ECG or Holter monitoring (Koch et al., 2022; Lundell and Ojanen, 2023; Lundell et al., 2020; Lundell et al., 2021; Rajdeep et al., 2014; Stelmaszczyk et al., 2019).

Compared with physiological diving responses, relatively little is known about autonomic regulation during decompression sickness (DCS). Experimental studies in animal models demonstrate substantial alterations in autonomic nervous system activity during DCS, including impaired autonomic modulation during neurological injury (Bai et al., 2009) and distinct autonomic signatures preceding cardiopulmonary DCS (Bai et al., 2013; Bai, 2011). Human data remain limited and consist primarily of isolated clinical observations and experimental studies of decompression stress (Schirato et al., 2018; Schmitz, 2025).

The objective of this study was therefore to describe heart rate variability patterns in a series of divers presenting with neurological decompression sickness prior to hyperbaric oxygen therapy, using time-domain, frequency-domain, and nonlinear HRV metrics.

## Case reports

We report eight adult divers with neurological decompression sickness who met both clinical inclusion criteria and predefined ECG signal-quality criteria for HRV analysis and were evaluated at our hyperbaric facility between the 1^st^ of February and the 9^th^ of December 2025, with full symptomatic resolution after a single hyperbaric oxygen therapy (HBOT) session, as summarized in **Table 1**. No significant past medical history was reported by any diver and none of the divers reported chronic medication use. Diagnosis of NDCS was established clinically by physicians experienced in diving medicine, based on dive exposure, symptom evolution, neurological findings, and response to hyperbaric oxygen therapy, after exclusion of alternative diagnoses when appropriate. Long-term neurological follow-up was not systematically available. All patients received hyperbaric oxygen therapy following the USN TT6 within 60 minutes of their arrival to the Hyperbaric Service and were evaluated 24 and 48 hours after the treatment to ensure symptomatic resolution.

**Table 1:** Summary of eight consecutive analyzable cases of neurological decompression sickness treated with a single session of hyperbaric oxygen therapy.

Case	Age	Gender	Gas used	Symptoms	Time to treatment	Imaging
1	26	Male	Air	Left paresthesia, vertigo, mild weakness	15 h	Chest X ray normal
2	42	Male	Air / surface-supplied air	Left hemiparesis, hypotension, bradycardia	72 h	Atelectasis
3	53	Male	EAN32	Right leg monoplegia, cutis, chest pain	40 h	Chest X ray normal
4	29	Male	Air / compressor-supplied air	Severe Inner Ear Decompression	30 h	Cerebral CT Scan normal
5	55	Female	EAN32	Right Lower Extremity Monoplegia	112 h	None
6	35	Male	EAN32	Vestibular Dysfunction and Left Upper Extremity Monoplegia	68 h	Chest X ray normal
7	39	Female	Air	Left hemiparesis with Diplopia	25 h	None
8	58	Female	Air	Right lower extremity monoplegia with amnesia	20 h	Cerebral CT Scan normal

### Case 1 – Cerebral and inner ear symptoms after a single recreational dive

A healthy 26-year-old recreational male diver (10 lifetime dives) completed a 60-min air dive to 20.8 msw without a safety stop. Within 5 min of surfacing, he developed transient cutis marmorata of both upper limbs, resolving spontaneously within 6 h. One hour post-dive, he reported vertigo, lightheadedness, headache, vague left abdominal discomfort, and left-sided paresthesias of the arm, leg, chest, abdomen, and back, with mild left upper-limb weakness.

Fifteen hours later he presented alert (GCS 15) with a right-sided Romberg sign and mild weakness of the left biceps and forearm flexors (4/5). Chest radiograph was normal. Treatment with a U.S. Navy Treatment Table 6 (USN TT6) led to complete resolution of symptoms by the end of the session, and he remained asymptomatic at 24- and 48-h follow-up.

### Case 2 – Hemiparesis after surface-supplied commercial diving

A 42-year-old professional surface-supplied male diver, performing 5–10 dives daily for 15 days, on the day of the event conducted a 60-min dive to ~30 msw for net repair. Shortly after surfacing, he developed left hemiparesis, vertigo, right facial palsy, bilateral hip and shoulder pain, hypotension, and bradycardia.

Three days later he presented to a hyperbaric facility with persistent left hemiparesis, musculoskeletal pain, and diffuse 2–4 cm bluish-brown macules on the trunk and upper limbs, compatible with cutaneous DCS. He was alert (GCS 15), hemodynamically stable, with monoparesis of the left upper limb (biceps and triceps 3–4/5) and a right-sided Romberg sign. Brain CT was normal; thoracic CT showed atelectasis without pneumothorax. D-dimer was moderately elevated. After a USN TT6, he was asymptomatic at 24- and 48-h review.

### Case 3 – Monoplegia and chest symptoms after repetitive diving

A 53-year-old male divemaster (~380 lifetime dives) completed 3–4 EAN32 dives per day over six consecutive days on a live-aboard, to a maximum depth of 32.7 msw. On day 5 he developed a transient abdominal rash, which recurred on day 6 with diffuse chest and neck pain and mild dysphonia. Four hours of surface oxygen did not relieve symptoms. On return to shore, he noted persistent abdominal cutis marmorata, right-sided chest discomfort, and reduced voice projection.

He presented about 40 h after the last dive with right lower-limb weakness (hip flexors, quadriceps, and extensors 4/5); Romberg sign, ECG, and chest X-ray were unremarkable. A single USN TT6 produced full symptom resolution within 20 min. He remained asymptomatic at 24 and 48 h. Laboratory tests (CBC, D-dimer, troponin, pro-BNP) and contrast transthoracic echocardiography showed no abnormalities or right-to-left shunt.

### Case 4 – Severe inner ear decompression sickness in an artisanal compressor diver

A 29-year-old male artisanal compressor diver presented after two days of intensive diving (three dives to 30 m on day 1, followed by two dives to 40 m on day 2, including one of 140 minutes). Approximately 10 minutes after his second dive on the second day he developed left knee pain and bilateral shoulder pain. He attempted in-water recompression at 10 msw, but was forced to perform an emergency ascent due to a hose malfunction; upon surfacing, he developed left-sided vertigo. A second in-water recompression attempt failed due to mask problems. Symptoms progressively worsened, evolving to severe left-sided vertigo with inability to walk, complete left-sided hearing loss, nausea, vomiting, and shoulder pain. On initial evaluation, 30 h after surfacing, he was alert (GCS 15), normotensive, with a positive Romberg (immediate left deviation), comparative left-sided deafness, left horizontal nystagmus, and inability to ambulate, without motor deficits in the extremities.

### Case 5 – Right lower extremity monoplegia preceded by recurrent cutaneous manifestations

A 55-year-old female experienced diver (Master Scuba Diver, Nitrox certified, 570 lifetime dives) presented 112 hours after surfacing following a liveaboard trip. Over multiple dive days on EAN32, she experienced recurrent cutaneous DCS (pruritic skin rash on the left breast and abdomen), following an accidental rapid ascent from 12 to 3 msw. Symptoms resolved spontaneously or during subsequent dives on multiple occasions. On her fifth dive day, after three dives (maximum 27 msw), she developed extreme fatigue and phosphenes, which resolved with rest. On the sixth dive day, a single dive to 27 msw triggered recurrence of the abdominal rash, which resolved with 1 hour of surface oxygen; however, back pain subsequently developed and responded to an additional hour of oxygen. On presentation, neurological examination revealed a negative Romberg but significant right lower extremity weakness (hip flexor, gluteus maximus, hamstrings, extensors, and gastrocnemius graded 3/5; quadriceps 4/5), with normal upper extremity strength.

### Case 6 – Vestibular dysfunction and left upper extremity monoplegia in a professional dive instructor

A 35-year-old male dive instructor presented 68 hours after surfacing, following seven days of three-dives-per-day on EAN32 (depths ranging 75–104 fsw). On his day 5, after his third dive, he developed a persistent bilateral abdominal skin rash (cutis marmorata) without other symptoms, which did not resolve spontaneously. He continued diving on the last day without incident. On presentation, he additionally reported intermittent right-sided chest discomfort during inhalation underwater, present for several months. Neurological examination revealed a positive Romberg to the left and left upper extremity weakness (deltoids and biceps 4/5, forearm flexor 3/5, forearm extension 4/5), with normal lower extremity strength.

### Case 7 – Left hemiparesis with diplopia following repetitive recreational diving

A 39-year-old female recreational diver (AOWD, 40–50 lifetime dives) presented 25 hours after completing three consecutive dives (maximum depths 22–28 m, total bottom time ~158 minutes). She developed epigastralgia, fatigue, diplopia, bilateral elbow pain, and generalized muscle weakness. Neurological examination revealed a positive Romberg (immediate left deviation), right hemiparesis affecting primarily the lower extremity (gluteus maximus, quadriceps, and hamstrings graded 3/5), and mild upper extremity weakness (deltoids and biceps 4/5 on the right).

### Case 8 – Right lower extremity monoplegia with amnesia

A 58-year old female recreational diver (Rescue Diver, 78 lifetime dives) presented 20 hours after ten consecutive dives, two per day, all on air and within non decompression limits. About 10 minutes after her last dive she presented skin rash on her abdomen. About 2 hours after the dive she presented an episode of amnesia without loss of consciousness for 20 minutes. Cerebral CT Scan and Chest X-ray were reported as normal. Physical examination showed right lower-extremity monoplegia, abdominal hyperesthesia, psychomotor slowing, impaired abstract thinking, and confusion. All symptoms resolved after a single hyperbaric treatment session.

## Methodology

### Study design and setting

This case series describes eight adult divers diagnosed with neurological decompression sickness (NDCS) who were evaluated at a tertiary hyperbaric medicine center in Costa Rica between 1^st^ of February and 9^th^ of December 2025. All patients presented with acute or subacute neurological symptoms following scuba diving and underwent standardized clinical evaluation and neurophysiological monitoring prior to hyperbaric oxygen therapy (HBOT).

### Patient identification, routine ECG monitoring, and analytical cohort

This study was designed as a retrospective descriptive case series. The year 2025 was predefined as the review period for identifying eligible patients with neurological decompression sickness evaluated at our hyperbaric medicine center. At our institution, all patients treated for decompression sickness routinely undergo continuous ECG monitoring before, during, and after hyperbaric oxygen therapy as part of the standard clinical protocol to ensure cardiac and hemodynamic stability during treatment. Therefore, the ECG recordings analyzed in this study were obtained as part of routine clinical monitoring rather than as a prospective research intervention.

During the 2025 retrospective review period, nine adult divers with clinically diagnosed neurological decompression sickness underwent pre-HBOT ECG monitoring and were initially considered for HRV analysis. One patient, evaluated chronologically between the current Cases 3 and 4, was excluded from the final HRV analysis because of significant atrial dysrhythmia with excessive irregular RR intervals. This exclusion was applied after inspection of the ECG-derived RR interval series and signal-quality metrics, because the rhythm disturbance precluded reliable normal-to-normal interval reconstruction and could have substantially biased time-domain, frequency-domain, and entropy-based HRV metrics.

The final analytical cohort therefore consisted of eight consecutive analyzable patients who fulfilled both clinical eligibility criteria and ECG signal-quality criteria for HRV analysis. No additional patients from the 2025 review period were excluded because of missing clinical information, unavailable pre-HBOT ECG data, inadequate recording duration, insufficient raw ECG quality, or incomplete HRV processing. Thus, the final cohort represents the consecutive analyzable NDCS cases from the predefined 2025 retrospective review period after exclusion of one patient with clinically significant atrial dysrhythmia.

### Clinical severity scoring

Clinical severity was assessed using three previously published decompression sickness scoring systems. The Boussuges score is a neurological prognostic score developed for spinal cord decompression sickness and incorporates the distribution and severity of neurological deficits (range 1–22, higher scores indicating greater severity) (Blatteau et al., 2011). The Perceived Severity Index (PSI) is a probabilistic severity classification system based on symptom burden and estimated DCS severity ranging from 1 to 6 (Howle et al., 2017). The MEDSUBHYP score is a clinical severity scale developed to quantify decompression illness severity across neurological and systemic manifestations (range 0–21, higher scores indicating greater severity) (Blatteau et al., 2011). Scores were assigned based on the initial neurological examination performed prior to HBOT. Clinical severity scores are summarized in **Table 2**.

**Table 2:** Comparison of decompression sickness (DCS) severity across eight clinical cases using three established grading systems.

Case	Boussuges score (1–22)	PSI (1-6)	MEDSUBHYP score (0-21)
Case 1	4	3	7
Case 2	6	1	9
Case 3	6	2	8
Case 4	12	1	10
Case 5	12	1	11
Case 6	16	1	13
Case 7	15	1	14
Case 8	10	1	9

### Decompression stress

Dive exposure was summarized descriptively using the available clinical dive history and, when available, reconstructed dive-profile information. Breathing gas for the relevant index or repetitive dive exposure is reported in **Table 1**. Estimated decompression exposure was characterized using a custom Bühlmann ZHL-16 implementation, reporting the maximum fraction of the allowable inert-gas gradient reached during the reconstructed profile and the tissue half-time compartment in which this maximum occurred. These decompression variables were used only as descriptive indicators of exposure and were not treated as validated predictors of clinical severity. Estimated decompression exposure variables are summarized in **Table 3**. Full modeling details are provided in **Supplementary Methods S1**.

**Table 3:** Dive exposure characteristics and estimated decompression stress using the Bühlmann ZHL-16 model.

Case	Days of consecutive dives	% of maximum gradient factor	Compartment of maximum Bühlmann gradient fraction (tissue half-time, min)
Case 1	1	52%	18.5
Case 2	15	79%	18.5
Case 3	6	72%	18.5
Case 4	10	139%	53
Case 5	2	56%	37
Case 6	7	84%	18.5
Case 7	2	63%	12.5
Case 8	10	54%	12.5

### ECG monitoring and HRV analysis

Continuous electrocardiographic monitoring was performed for 20 minutes prior to hyperbaric oxygen therapy (HBOT) in a quiet, temperature-controlled room with patients in the supine position. Recordings were obtained during spontaneous breathing without paced respiratory control or respiratory monitoring. Due to the acute clinical setting, factors such as recent food intake, caffeine consumption, circadian variability, pain, psychological stress, and medication exposure could not be fully standardized across patients.

**Figure 1:**
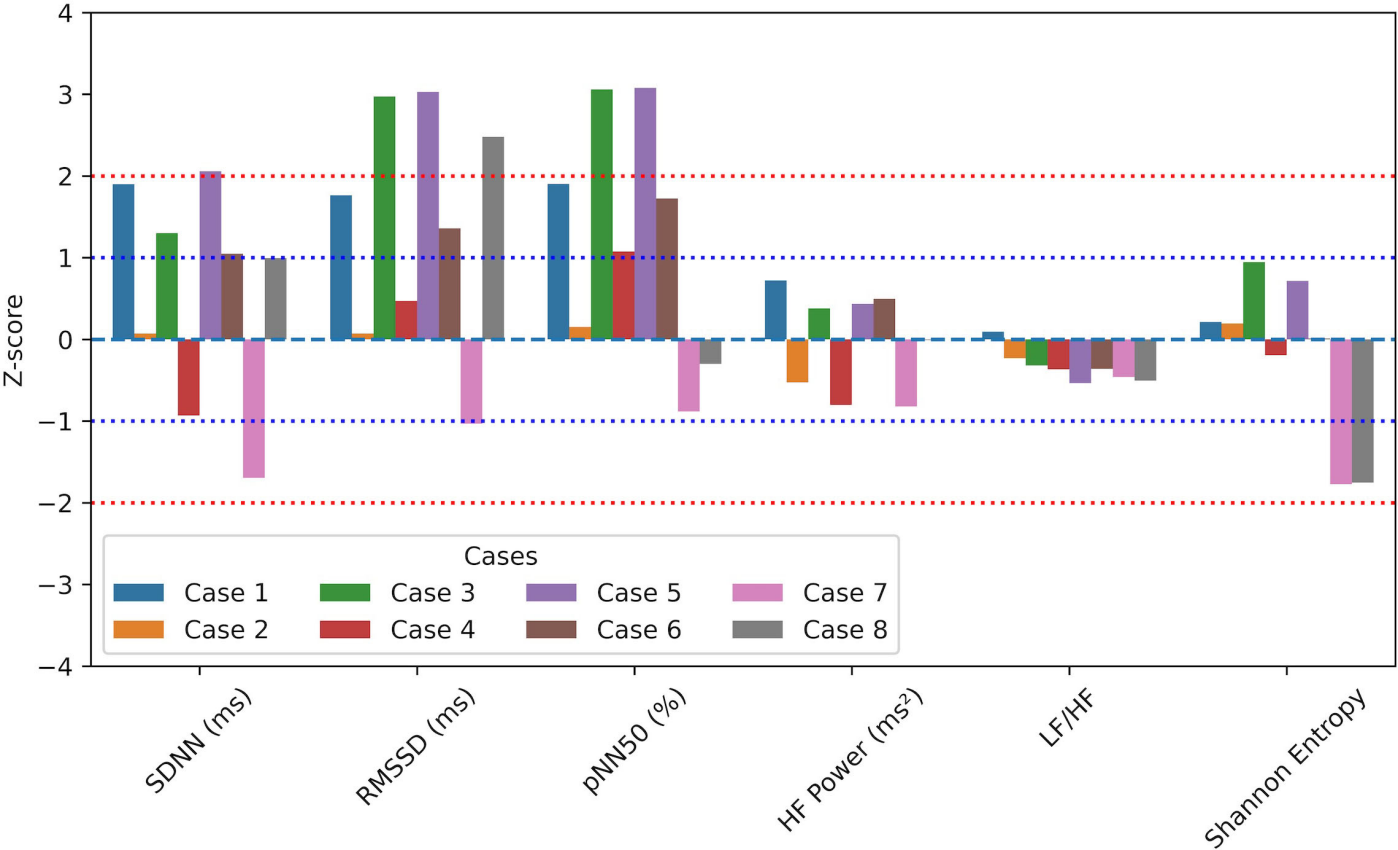
Z-score normalized heart rate variability (HRV) profiles for eight cases of neurological decompression sickness (NDCS). The bar chart illustrates deviations from normative reference values (Z = 0, blue dashed line). The blue dotted lines mark one standard deviation (Z = ± 1), and the red dotted lines mark two standard deviations (Z = ± 2). Metrics include SDNN, RMSSD, pNN50, HF Power, LF/HF, and Shannon Entropy. Age- and sex-specific reference means and standard deviations used for Z-score computation were obtained from Voss et al. (2015) and are reported in **Supplementary Table S2**. Entropy-based Z-scores are exploratory because entropy estimates are method-dependent and the reference entropy metric is not identical to the histogram-based Shannon entropy used in the present analysis.

ECG acquisition was performed using a Mindray BeneVision N19 monitor. The sampling configuration provided a temporal resolution of approximately 2 ms, sufficient for accurate R-peak detection and short-term HRV analysis. Raw ECG traces were exported for offline processing using a custom Python-based analytical pipeline (Python version 3.14.3) utilizing the NeuroKit2 and SciPy libraries, following international standards for HRV analysis and interpretation (Task Force of the European Society of Cardiology and the North American Society of Pacing and Electrophysiology, 1996). From each 20-minute pre-HBOT ECG recording, an approximately 10-minute analyzable segment was selected for HRV analysis. The target duration was 10 minutes; however, the final analyzed duration varied slightly across cases because terminal artifacts or non-analyzable edge intervals were excluded during RR/NN reconstruction. The final analyzed duration for each case is reported in **Supplementary Table 1**. All HRV values reported in **Table 4** and all Z-scores shown in **Figure 1** were computed from these final approximately 10-minute analyzable segments.

**Table 4:** Absolute HRV metrics.

Case	SDNN (ms)	RMSSD (ms)	PNN50 (%)	HF Power (ms^2^)	LF/HF	SD1	SD2	Shannon entropy
Case 1	81.5	66.3	43.3	1056.4	2.09	46.9	105.3	3.09
Case 2	47.1	35.3	16.3	223.0	1.30	24.9	61.8	3.08
Case 3	52.2	53.2	41.2	510.4	1.04	37.7	63.6	2.90
Case 4	28.3	42.6	30.52	40.6	0.97	30.2	26.2	2.89
Case 5	59.6	62.0	41.4	537.8	0.57	43.8	72.0	2.87
Case 6	65.5	58.8	40.6	907.5	0.98	41.6	82.7	2.99
Case 7	12.4	15.8	0.4	27.0	0.74	11.2	13.4	2.21
Case 8	45.1	54.7	3.9	321.1	0.63	38.7	50.8	1.59

Segment selection was performed manually before HRV metric computation, using the same technical rule in all cases: continuity of the ECG trace, relative signal stability, absence of major movement or signal-loss artifacts, and the lowest visible artifact burden within the available pre-HBOT recording. The segment was not selected on the basis of the resulting HRV values. The analyst was not blinded to the clinical presentation, because this was a retrospective clinical case series and the ECG recordings were reviewed together with the clinical record. All selected segments were obtained after arrival to the hyperbaric service and before HBOT initiation, during routine pre-treatment monitoring; exact segment start times relative to arrival and HBOT initiation were not systematically recorded.

R-peaks were detected from the ECG recordings using a modified Pan–Tompkins-based offline processing pipeline. Raw RR interval tachograms were first generated from the detected R peaks and inspected as part of the signal-quality control procedure. Ectopic or artifact intervals were identified using predefined physiological limits and local deviation criteria. Specifically, RR intervals <300 ms or >2000 ms were classified as nonphysiological. In addition, RR intervals showing >20% relative deviation from the surrounding local median within a moving local window were classified as ectopic/artifact intervals. The resulting classifications were verified by visual inspection of the raw RR tachograms and corrected NN tachograms.

Internal artifact intervals surrounded by valid RR intervals were corrected by interpolation before HRV computation. Terminal artifact intervals located at the beginning or end of the analyzed series, when not reliably bracketed by valid neighboring intervals, were excluded rather than interpolated to avoid edge-related interpolation distortion. For each case, the number of detected R peaks, NN intervals analyzed, ectopic/artifact intervals, and intervals corrected by interpolation were documented. Only recordings with <5% ectopic/artifact contamination were retained for final HRV analysis. Per-case signal-quality metrics, raw RR tachograms, and corrected NN tachograms are provided in **Supplementary Table 1**, **Supplementary Figure 1**. The excluded patient with significant atrial dysrhythmia is shown separately in **Supplementary Figure 2**.

Time-domain metrics included mean RR interval, SDNN, RMSSD, and pNN50. Frequency-domain analysis included high-frequency (HF) power and the LF/HF ratio. Conventional frequency band limits were defined as 0.04–0.15 Hz for low frequency (LF) and 0.15–0.40 Hz for high frequency (HF). Prior to spectral analysis, RR interval series were interpolated and resampled at 4 Hz and linearly detrended. Power spectral density was estimated using Welch’s method with a maximum segment length of 256 samples and 50% overlap.

Nonlinear analysis included histogram-based Shannon entropy and two-dimensional Poincaré plot analysis. Shannon entropy was calculated from the probability distribution of RR intervals using a histogram-based approach with 20 bins. No phase-space embedding or delay reconstruction parameters were applied, as the entropy analysis was performed directly on the one-dimensional RR interval distribution. Two-dimensional Poincaré plots were constructed by plotting each RR interval (RRn) against the subsequent interval (RRn+1). From these plots, short-term variability (SD1, transverse axis) and long-term variability (SD2, longitudinal axis) were calculated using standard geometric formulations to characterize attractor morphology and variability dispersion.

HRV results were expressed both as absolute values and as exploratory standardized Z-scores relative to age- and sex-specific reference values reported by Voss et al. (2015). That study provides short-term HRV reference distributions from 5-minute ECG recordings obtained in healthy subjects from the KORA S4 cohort, stratified by sex and age categories. For each patient, the corresponding reference group was selected according to sex and age decade. Z-scores were calculated as: Z = (observed value − reference mean)/reference standard deviation.

The reference mean and standard deviation used for each metric and each case are reported in **Supplementary Table 2**. Z-score normalization was used only as a descriptive approach to contextualize individual HRV values against published healthy reference distributions and was not used for formal statistical inference or diagnostic classification. Because the reference values from Voss et al. were derived from 5-minute ECG recordings, whereas the present analysis used approximately 10-minute analyzable pre-HBOT segments, standardized deviations were interpreted cautiously.

Entropy-based Z-scores were interpreted with particular caution because entropy estimates are method-dependent. The reference entropy metric available in Voss et al. is not identical to the histogram-based Shannon entropy calculated in the present analysis; this distinction is detailed in **Supplementary Table 2**. Therefore, entropy Z-scores were used only as approximate exploratory comparators rather than as normative classifications.

## Results

Eight male and female divers presented with focal neurological deficits consistent with neurological decompression sickness (NDCS), including monoplegia, hemiparesis, and vestibular dysfunction. Clinical severity varied from moderate to severe across the cohort based on composite scoring (Boussuges, PSI, and MEDSUBHYP).

Decompression exposure reconstruction using the Bühlmann ZHL-16 model yielded maximum Bühlmann gradient fractions ranging from 52% to 139%, with the highest value observed in Case 4. All subjects achieved complete symptom resolution following a single hyperbaric oxygen therapy (HBOT) session using the USN TT6 protocol.

### Time-domain HRV metrics

**Figure 2:**
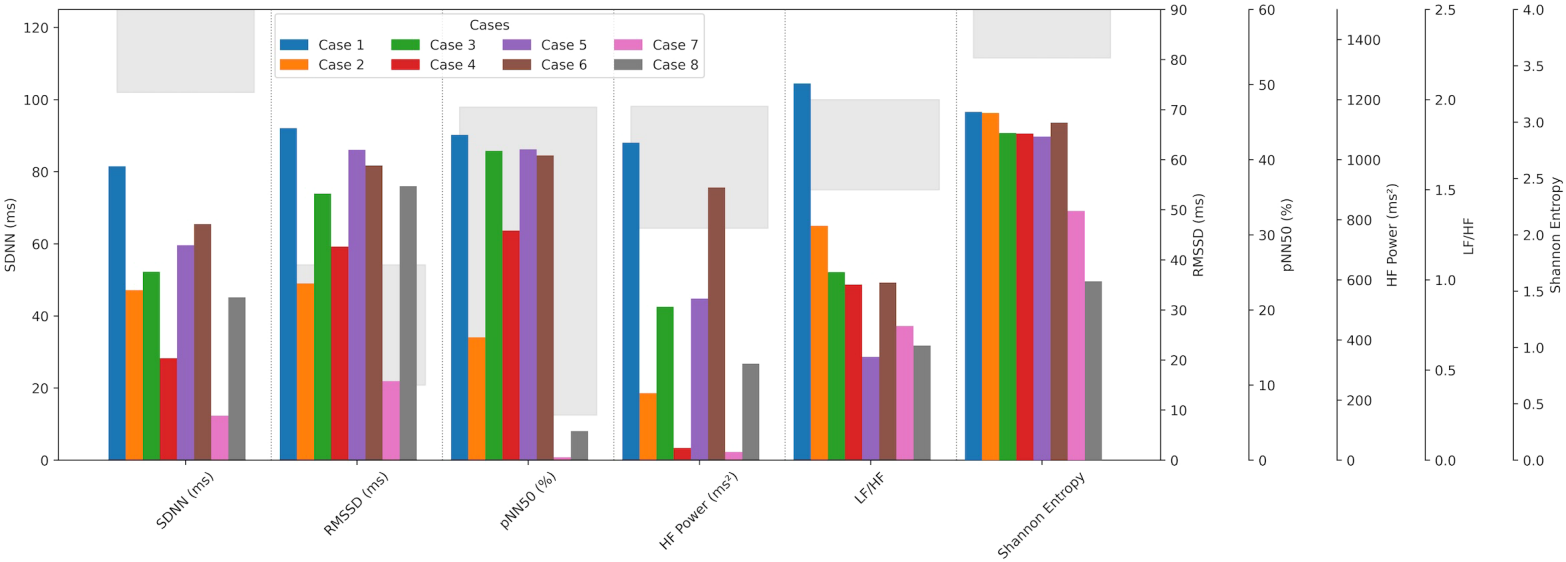
Absolute heart rate variability (HRV) metrics across eight cases of neurological decompression sickness (NDCS). The figure displays absolute values for time-domain, frequency-domain, and nonlinear/descriptive rhythm-structure metrics for each of the eight subjects prior to hyperbaric oxygen therapy. Gray shaded regions represent normative reference ranges for a healthy adult population (Task Force of the European Society of Cardiology and the North American Society of Pacing and Electrophysiology, 1996). Time-domain metrics: SDNN (ms), RMSSD (ms), and pNN50 (%) demonstrate varied absolute variability across the cases, with some subjects exhibiting values significantly outside the normative gray bands. Frequency-domain metrics: HF Power (ms^2^) and the LF/HF ratio reflect the distribution of spectral power. Nonlinear metrics: Shannon Entropy.

Time-domain HRV metrics did not show a uniform response pattern across the cohort. SDNN and RMSSD varied substantially between cases, with elevated values in some patients and reduced or near-reference values in others. This supports marked interindividual heterogeneity rather than a single time-domain HRV phenotype in NDCS. Similarly, pNN50 remained low in Cases 4, 7, and 8, suggesting attenuation of short-term beat-to-beat variability in these cases (**Figures 1**, **2**).

### Frequency-domain HRV metrics

Frequency-domain HRV metrics also showed substantial interindividual variability. HF power was highest in Case 1 and was also relatively high in Case 6, whereas Cases 4, 7, and 8 showed substantially lower HF power. LF/HF ratio values were lower than the corresponding age- and sex-specific reference values in several cases, particularly Cases 4 through 8. Because respiration was spontaneous and not monitored, HF power and LF/HF ratio were interpreted as descriptive frequency-domain indices rather than direct measures of parasympathetic or sympathovagal balance.

### Nonlinear HRV dynamics

**Figure 3:**
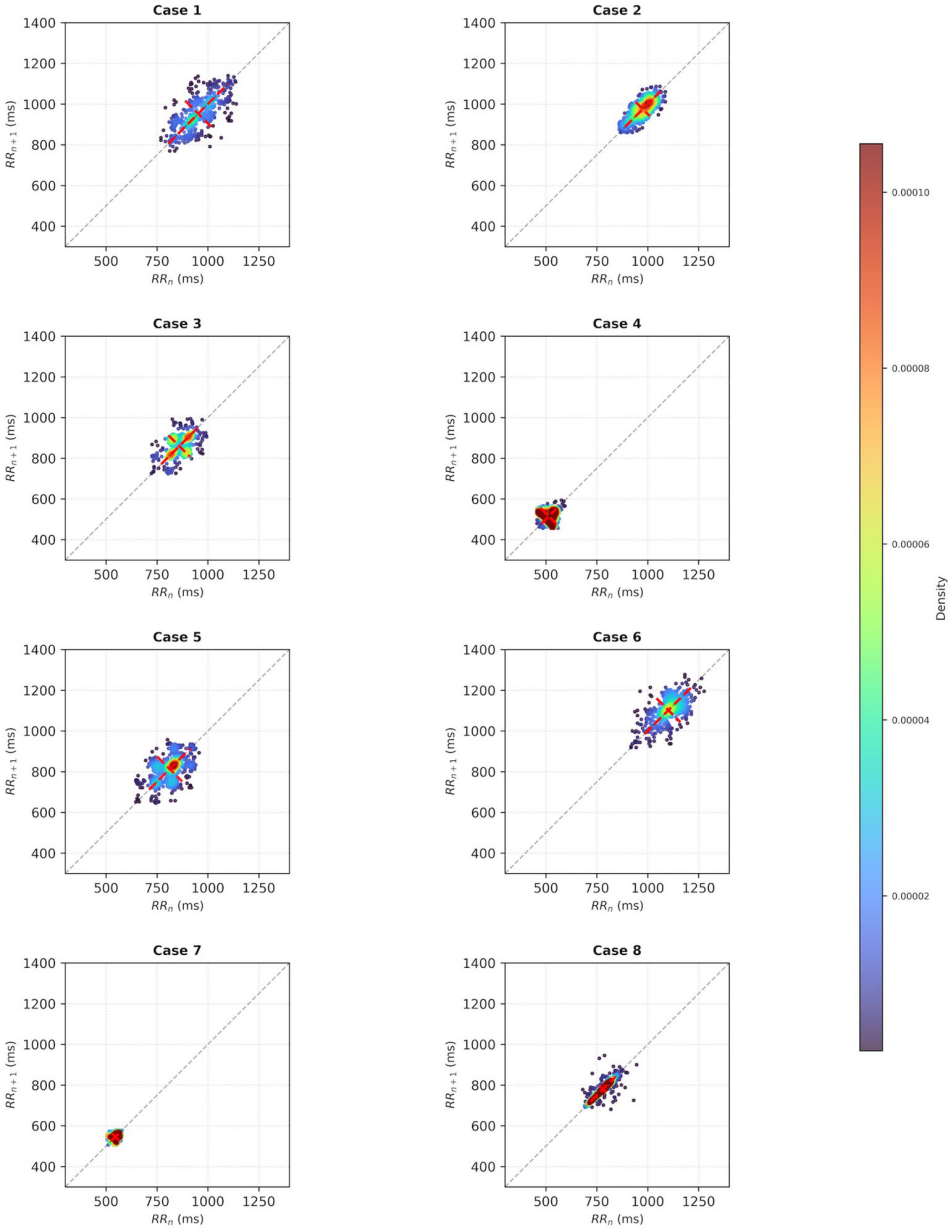
Two-dimensional Poincaré plots for cases 1–8. Each plot shows the relationship between consecutive RR intervals (RR_n_ vs. RR_{n+1}_), with the longitudinal and transverse axes representing SD2 and SD1, respectively. The plots illustrate the varying degrees of total variability and attractor geometry across the patient cohort prior to hyperbaric intervention.

Among the nonlinear descriptors, Shannon entropy was lower in a subset of cases, most clearly in Cases 7 and 8. Thus, entropy-based findings suggested heterogeneous rather than uniformly reduced distributional complexity of the NN interval series. These findings were interpreted as exploratory because entropy estimates are sensitive to preprocessing, binning strategy, segment selection, and the reference method used for comparison. Two-dimensional Poincaré plots further illustrated the variability in attractor geometry across cases (**Figure 3**).

## Discussion

This case series represents, to our knowledge, one of the few available human descriptions of HRV in NDCS. Given the rarity and clinical heterogeneity of neurological decompression sickness, this study should be interpreted as an exploratory case series intended to generate physiological hypotheses rather than establish definitive autonomic patterns.

Based on these eight cases of neurological decompression sickness (NDCS), two principal observations emerge regarding autonomic dysfunction as assessed by HRV. First, Shannon entropy showed a heterogeneous but clinically relevant pattern, with near-reference values in some cases and clear reductions in others, particularly Cases 7 and 8. Therefore, entropy did not identify a uniform cohort-wide abnormality, but it remained useful as an exploratory descriptor of NN interval distributional irregularity. Second, several conventional HRV indices (SDNN, RMSSD, HF) showed notable deviations from reference values. However, the direction and magnitude of these deviations varied considerably between individuals, and therefore no specific HRV pattern can be considered characteristic of DCS in this series.

Healthy diving studies consistently demonstrate dynamic autonomic modulation during immersion and recovery. In general, immersion is associated with increased parasympathetic activity and enhanced HRV indices consistent with the classical diving response, whereas psychologically stressful, cold-water, deep, or exercise-intensive dives may produce mixed sympathetic-parasympathetic activation patterns and reduced vagally mediated HRV indices (Schipke et al., 2001; Lafère et al., 2021; Schaller et al., 2021; Flouris and Scott, 2009). Most post-dive autonomic changes described in healthy divers normalize within minutes to hours after surfacing, although repeated or physiologically demanding exposures may lead to delayed recovery and reduced HRV complexity (Berry et al., 2017; Schirato et al., 2018).

Importantly, these physiological responses differ substantially from the present cohort, which consisted of patients evaluated during the clinical presentation of neurological decompression sickness between 15 and 112 hours after the index dive. Therefore, the HRV alterations observed in this study should not be interpreted as representations of normal post-dive physiology, but rather as autonomic patterns observed during the subacute clinical phase of NDCS, potentially influenced by neurological injury, pain, stress, inflammation, recovery dynamics, and pre-hospital interventions.

Experimental animal studies suggest that decompression sickness itself is associated with substantial disturbances in cardiac autonomic control. In swine models, neurological DCS has been associated with reductions exceeding 55% in both sympathetic and parasympathetic modulation, indicating global impairment of autonomic regulation rather than simple sympathovagal imbalance (Bai et al., 2009). In contrast, cardiopulmonary DCS in a porcine saturation-dive model is preceded by a transient simultaneous increase in both sympathetic and parasympathetic activity approximately 20 minutes before clinical onset. At symptom onset, parasympathetic activity remains elevated while sympathetic modulation decreases (Bai et al., 2013; Bai, 2011). These findings suggest that distinct autonomic signatures may characterize different DCS phenotypes and that advanced HRV analyses, including principal dynamic mode (PDM) approaches, may help differentiate these conditions and potentially provide early warning markers of cardiopulmonary DCS (Bai et al., 2009, Bai et al., 2013; Bai, 2011).

Human data remain limited. A recent case report of neurological DCS associated with sinoatrial dysfunction described a biphasic autonomic response characterized by transient higher vagally associated HRV indices during hyperbaric oxygen therapy followed by altered nonlinear HRV measures during recovery (Schmitz, 2025). Although limited to a single observation, this report illustrates the potential heterogeneity and temporal variability of autonomic responses in decompression illness.

Similarly, in a simulated dive experiment, healthy subjects exhibited a post-dive increase in LF power without significant increases in SDNN, RMSSD, or HF compared with controls. This pattern was interpreted as reflecting subclinical decompression-related endothelial or physiological stress in the absence of overt DCS. In contrast, one volunteer who developed clinical DCS in the same study demonstrated increased global HRV (SDNN) and elevated vagally mediated HF power, accompanied by marked inflammatory activation and microparticle release. These findings suggest a qualitatively different and dysregulated autonomic response in overt decompression injury compared with the LF-dominant response observed in asymptomatic divers (Schirato et al., 2018).

Collectively, available animal and human data indicate that decompression sickness is associated with substantial disturbances in autonomic regulation. These findings support the concept of autonomic dysfunction as an important component of DCS pathophysiology and suggest that HRV-derived metrics, particularly those derived from nonlinear and dynamic analyses, may represent promising—although still exploratory—biomarkers for early detection and phenotyping of decompression injury (Bai et al., 2009; Schmitz, 2025; Bai et al., 2013; Bai, 2011).

In the present case series, HRV findings also showed a tendency toward increased overall variability as reflected by SDNN, together with increased vagally associated indices such as RMSSD and HF, considering a z-score normalization of the absolute values. This observation is broadly consistent with previously reported experimental and clinical findings.

At the same time, entropy-based measures did not show a uniform reduction across all cases. Rather, lower entropy values were most evident in Cases 7 and 8, while other cases showed near-reference or only mildly reduced values. Therefore, the present findings should be interpreted as heterogeneous alterations in NN interval distributional structure, rather than as evidence of a consistent cohort-wide reduction in NN interval distributional irregularity.

An alternative explanation for the combination of increased conventional HRV variability indices together with lower entropy-based values involves residual ectopic-beat contamination or preprocessing artifacts, as distorted RR interval dynamics may artificially increase short-term variability while simultaneously degrading entropy-based measures. Several precautions were taken to minimize this effect, including adaptive artifact detection, exclusion of recordings with >5% ectopic/artifact contamination, and NN interval reconstruction prior to analysis. Nevertheless, given the sensitivity of nonlinear HRV metrics to signal preprocessing and the acute clinical setting of the recordings, the possibility that some of the observed reductions in complexity partially reflect residual signal contamination cannot be fully excluded.

Although elevated HRV is often interpreted as a marker of favorable autonomic flexibility, increased variability in pathological contexts may also reflect unstable or dysregulated autonomic control, particularly when accompanied by lower entropy-based values (Hager et al., 2025; Ernst, 2017).

One speculative interpretation of the observed dissociation between conventional HRV variability and lower entropy-based values is that NDCS may involve transient disruption of integrated autonomic regulation. Potential mechanisms could include decompression-related endothelial dysfunction, inflammatory activation, microvascular bubble effects, or altered central autonomic processing, although the present study was not designed to evaluate these mechanisms directly.

In healthy systems, cardiac rhythm emerges from the coordinated interaction of multiple regulatory loops—including central autonomic networks, baroreflex control, and peripheral feedback mechanisms—producing complex and adaptable heart rate dynamics.

Heart rate variability provides only an indirect assessment of autonomic regulation through cardiac rhythm dynamics and therefore represents a single effector output of the autonomic nervous system. In acute pathological states, HRV may also be influenced by factors such as respiratory irregularity, ectopic activity, pain, psychological stress, and signal preprocessing limitations.

Future prospective studies of decompression illness could benefit from combining HRV with additional non-invasive autonomic monitoring modalities. Dynamic pupillometry provides quantitative assessment of pupillomotor function and has been proposed as a practical autonomic testing tool, with altered pupillary indices reported in patients with autonomic dysfunction (Muppidi et al., 2013) Pupillary hippus has also been investigated as a potential autonomic biomarker using spectral and complexity-based approaches (Turnbull et al., 2017; Rizzuto et al., 2025). In the diving context, pupillometry could be explored as a complementary autonomic measure in future studies of immersion-related physiology.

An additional source of variability in the present series relates to the marked heterogeneity of the divers themselves, including differences in training background, cumulative diving exposure, professional experience, and diving practices. Such factors are known to influence baseline autonomic tone and physiological responses to immersion and decompression stress, potentially contributing to the interindividual variability observed in HRV patterns. Future prospective studies of decompression illness may benefit from more standardized multidimensional characterization of divers, including training intensity, exposure frequency, lifestyle factors, psychological stress, and diving-specific physiological adaptations, in order to improve subject stratification despite the inherently limited sample sizes typical of NDCS research.

In summary, this exploratory case series suggests that neurological decompression sickness may be associated with measurable but heterogeneous alterations in cardiac rhythm dynamics. Conventional HRV indices varied in both direction and magnitude, and entropy-based measures were clearly reduced only in a subset of patients, particularly Cases 7 and 8. These observations should be interpreted as hypothesis-generating rather than as evidence of a consistent HRV phenotype in NDCS.

## Limitations

This study has several limitations. The small sample size, observational design, single time-point HRV acquisition, and absence of matched asymptomatic post-dive controls preclude statistical inference and limit generalizability. Consequently, it cannot be determined whether the observed HRV alterations were specific to neurological decompression sickness (NDCS) or reflected broader post-dive physiology, acute stress responses, pain, recovery dynamics, pre-existing interindividual variability, or other confounding factors.

In addition, the interval between surfacing and HRV acquisition varied substantially across cases (15–112 hours), and some patients received supplemental oxygen prior to evaluation, both of which may independently influence autonomic modulation. Finally, the normative datasets used for HRV standardization were derived from healthy resting populations under different experimental conditions and therefore should be interpreted as exploratory descriptive references rather than definitive inferential comparators.

The HRV segment was selected manually from the available pre-HBOT recording, with a target duration of approximately 10 minutes. Although the same technical selection rule was applied to all cases and selection was performed before HRV metric computation, the analyst was not blinded to clinical severity and minor differences in final analyzable duration occurred after exclusion of non-analyzable edge or terminal artifact intervals. Therefore, residual segment-selection bias cannot be excluded.

The Z-score normalization relied on age- and sex-specific reference values derived from 5-minute recordings in healthy subjects, whereas the present HRV analysis used approximately 10-minute pre-HBOT clinical recordings; therefore, standardized deviations should be interpreted as exploratory rather than diagnostic.

Respiration was spontaneous and not monitored. Therefore, HF power and RMSSD were interpreted only as vagally associated indices rather than direct measures of parasympathetic predominance. Altered breathing patterns related to pain, vertigo, nausea, anxiety, or recent oxygen administration may have influenced these metrics.

## Conclusion

This exploratory case series suggests that neurological decompression sickness may be associated with measurable but heterogeneous disturbances in cardiac rhythm dynamics reflected by HRV metrics. Conventional HRV indices varied substantially across cases, while entropy-based measures were lower in some patients, particularly Cases 7 and 8, but not uniformly reduced across the cohort. These findings should be interpreted as hypothesis-generating and do not establish a specific HRV phenotype of NDCS. Larger prospective studies with standardized respiratory monitoring, predefined segment selection, and matched post-dive controls are required to determine whether HRV analysis can contribute to the physiological characterization or monitoring of decompression sickness.

## Data Availability

The datasets generated and analyzed during the current study are not publicly available because they contain clinical monitoring data from a small case series and may include potentially re-identifiable information. De-identified data may be made available by the corresponding author upon reasonable request, subject to applicable ethical, legal, and privacy considerations.
