# Supplementary material for: Heart rate variability findings in neurological decompression sickness: an exploratory case series
Source: Front Physiol. 2026 Jun 10;17:1826066. doi: 10.3389/fphys.2026.1826066 (PMC13290766; doi:10.3389/fphys.2026.1826066)
Supplement: Supplementary file 1 [file DataSheet1.pdf]

Supplementary Material

Supplementary Tables

Supplementary Table 1. ECG signal-quality metrics for HRV analysis.

Per-case quality-control summary for ECG-derived RR/NN interval series. Case 9 represents the excluded patient with significant atrial dysrhythmia and is shown separately for transparency.

Case	Analyzed duration (min)	Detected R peaks (n)	NN intervals analyzed (n)	Ectopic/artifact intervals (n)	Ectopic/artifact intervals (%)	Intervals corrected by interpolation (n)	Intervals corrected by interpolation (%)	Segments excluded before final analysis
Case 1	9.77	617	616	28	4.55	28	4.55	No
Case 2	9.89	612	611	2	0.33	2	0.33	No
Case 3	9.85	623	619	7	1.13	4	0.64	No
Case 4	10	1156	1146	6	0.52	6	0.52	No
Case 5	11	814	803	0	0	0	0	No
Case 6	10	548	538	22	4.09	22	4.09	No
Case 7	9.58	985	976	6	0.61	6	0.61	No
Case 8	9.86	357	356	17	4.78	17	4.78	No
**Excluded case**	7.62	608	606	299	49.26	298	49.09	1 terminal artifact interval(s) excluded

Note. Abbreviations: ECG, electrocardiography; HRV, heart rate variability; NN, normal-to-normal interval; RR, R-to-R interval. Ectopic/artifact intervals were identified using predefined physiological limits and local deviation criteria; internal artifact intervals were corrected by interpolation.

Supplementary Table S2. Reference values used for Z-score normalization of HRV metrics

Reference source: Voss et al. (2015), age- and sex-stratified short-term HRV reference values from 5-minute ECG recordings in healthy subjects. Values are presented as mean ± SD. pNN50 values from Voss are reported as proportions and are converted here to percentages to match the manuscript units.

Case	Age	Gender	Reference stratum	N	SDNN (ms)	RMSSD (ms)	pNN50 (%)	HF power (ms^2)	LF/HF	Shannon entropy reference
1	26	Male	25-34 y male	330	50.0 ± 20.9	39.7 ± 19.9	20 ± 17	133 ± 174	2.79 ± 3.20	3.15 ± 0.42
2	42	Male	35-44 y male	292	44.6 ± 16.8	32.0 ± 16.5	13 ± 15	89 ± 118	3.62 ± 3.73	2.95 ± 0.44
3	53	Male	45-54 y male	235	36.8 ± 14.6	23.0 ± 10.9	6 ± 8	41 ± 49	4.10 ± 3.48	2.62 ± 0.47
4	29	Male	25-34 y male	330	50.0 ± 20.9	39.7 ± 19.9	20 ± 17	133 ± 174	2.79 ± 3.20	3.15 ± 0.42
5	55	Female	55-64 y female	95	30.6 ± 12.4	21.4 ± 11.9	5 ± 8	35 ± 53	2.87 ± 3.32	2.51 ± 0.49
6	35	Male	35-44 y male	292	44.6 ± 16.8	32.0 ± 16.5	13 ± 15	89 ± 118	3.62 ± 3.73	2.95 ± 0.44
7	39	Female	35-44 y female	259	45.4 ± 20.5	35.4 ± 18.5	16 ± 17	121 ± 145	2.21 ± 2.16	3.05 ± 0.46
8	58	Female	55-64 y female	95	30.6 ± 12.4	21.4 ± 11.9	5 ± 8	35 ± 53	2.87 ± 3.32	2.51 ± 0.49

Notes. Reference strata were selected according to each diver’s age and sex. Voss et al. report HRV reference values for 25–34, 35–44, 45–54, 55–64, and 65–74 year age strata separately for females and males. SDNN, RMSSD, pNN50, HF power, and LF/HF values are taken from the linear HRV tables. The Shannon entropy reference shown here corresponds to Voss et al.’s symbolic-dynamics Shannon entropy (shannon_SD), which is the entropy metric in the source whose numerical scale is closest to the current manuscript’s Shannon entropy values. This metric differs from both Voss et al.’s time-domain histogram entropy (shannon_h) and the histogram-based Shannon entropy method used in the present manuscript.

Source details. The source study analyzed 5-minute ECG recordings in healthy subjects from the KORA S4 cohort, stratified by sex and age. The authors extracted tachograms from 5-minute ECG recordings, visually inspected them, applied adaptive filtering to obtain NN intervals, and then calculated time-domain, frequency-domain, and nonlinear indices. Values in this table come from Tables 5–8 of Voss et al. (2015).

Abbreviations: SDNN, standard deviation of normal-to-normal intervals; RMSSD, root mean square of successive differences; pNN50, percentage of successive NN intervals differing by more than 50 ms; HF, high-frequency power; LF/HF, low-frequency to high-frequency power ratio.

Supplementary Table S3. CARE checklist for the present exploratory case series

Note. CARE was applied as a reporting guide for this retrospective exploratory case series. Items unavailable because of retrospective design are explicitly identified.

CARE item	Reporting element	Manuscript/supplement location	Status	Case-series specific comment
1. Title	Identify as case report/series and main clinical phenomenon.	Title page	**Addressed**	Title identifies an exploratory case series on HRV findings in neurological decompression sickness.
2. Keywords	Include condition- and method-relevant keywords.	Title page	**Addressed**	Keywords include decompression sickness, HRV, autonomic nervous system, HBOT, and nonlinear dynamics.
3. Abstract	Summarize background, methods, results, and conclusion.	Abstract	**Addressed**	Abstract emphasizes heterogeneous HRV findings and avoids overstatement of consistent entropy or complexity reduction.
4. Introduction	Explain rationale and prior evidence.	Introduction	**Addressed**	Provides rationale for HRV assessment in NDCS and summarizes limited human/animal evidence.
5. Patient information	Report age/sex, history, dive exposure, symptoms.	Case reports; Table 1	**Addressed**	Demographics, dive exposure, breathing gas, symptoms, and clinical findings are reported.
6. Clinical findings	Describe relevant examination findings.	Case reports; Table 1	**Addressed**	Neurological and vestibular findings are described for each case.
7. Timeline	Provide chronological summary.	Case reports; Table 1	**Partial**	Time to HBOT is reported. No separate graphical timeline was prepared.
8. Diagnostic assessment	Describe diagnostic reasoning, tests, and limitations.	Case reports; Methods	**Partial**	NDCS diagnosis was clinical. Imaging/ancillary tests were performed when clinically indicated, not standardized across cases.
9. Therapeutic intervention	Describe intervention, protocol, timing, administration.	Case reports; Methods	**Addressed**	All patients received one USN TT6. Systematic tolerability and adverse-event data were not available because of the retrospective design.
10. Follow-up/outcomes	Report outcomes, follow-up, adverse events.	Case reports; Limitations	**Partial**	Clinical status at 24 and 48 h is reported. Longer-term follow-up data were unavailable.
11. Discussion	Discuss strengths, limitations, literature comparison, rationale.	Discussion; Limitations	**Addressed**	Discussion emphasizes heterogeneity and avoids causal or definitive claims regarding entropy-based complexity.
12. Patient perspective	Include patient perspective when available.	Not available	**Not available**	Patient perspectives were not systematically collected because of the retrospective design; none were inferred.
13. Informed consent	State whether consent was obtained.	Title page / confirmations	**Addressed**	Manuscript states written informed consent for clinical evaluation, HRV monitoring, and anonymized publication.

Abbreviations: CARE, CAse REport; DCS, decompression sickness; HBOT, hyperbaric oxygen therapy; HRV, heart rate variability; NDCS, neurological decompression sickness; USN TT6, U.S. Navy Treatment Table 6.

Supplementary Figures

Supplementary Figure S1. Raw and corrected tachograms for the analyzed ECG segments in the included cases.
For each case, the upper panel shows the raw RR tachogram derived from R peaks detected in the original ECG recording. Intervals classified as ectopic or artifact are marked with crosses. The lower panel shows the corrected NN tachogram after interpolation of internal artifact intervals; interpolated points are marked with circles. Vertical dashed lines indicate transitions between consecutive 60-second ECG image segments.

Figure S1a: Cases 1-4

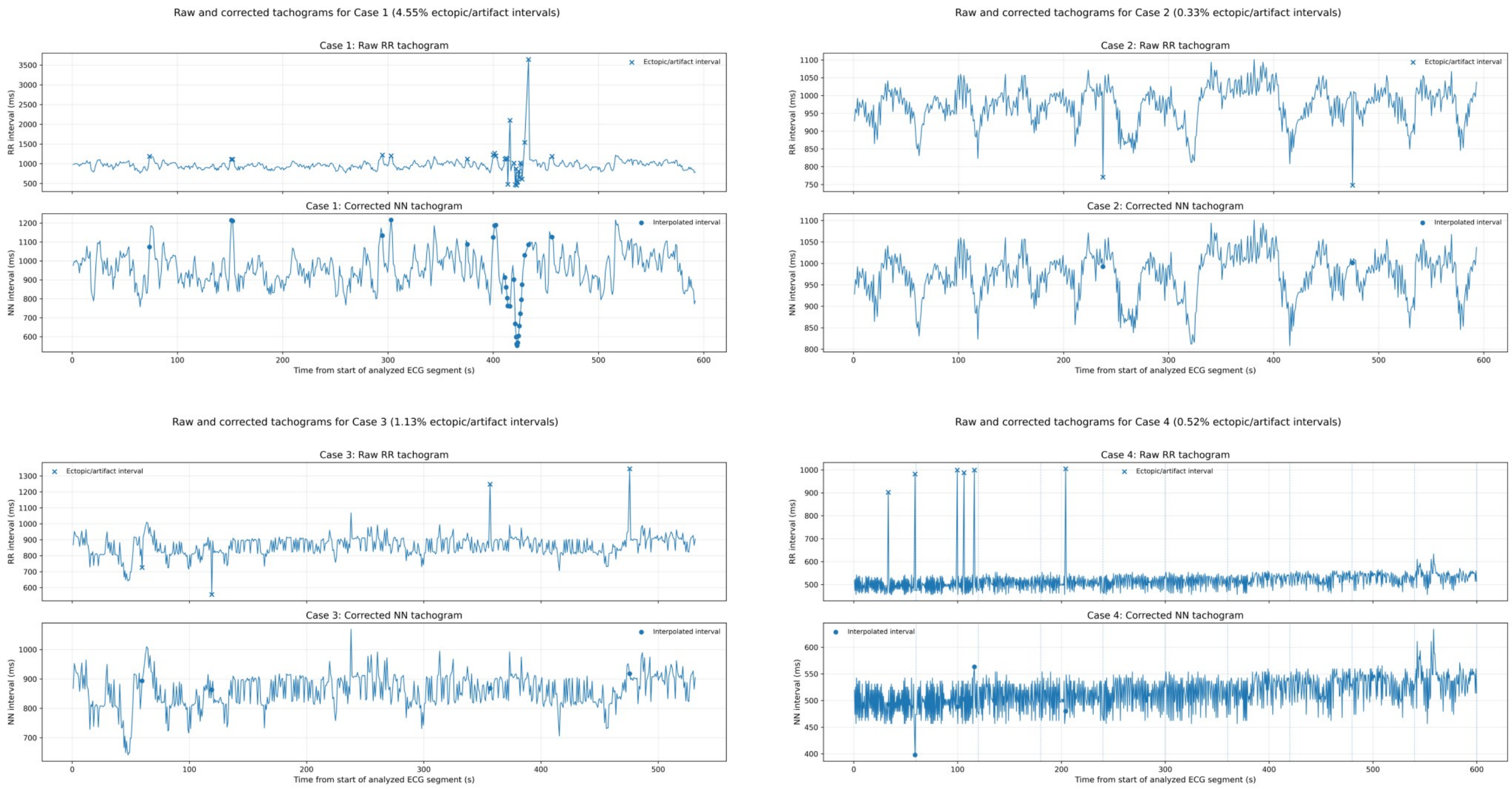

Figure S1a: Cases 5-8

Raw and corrected tachograms for Case 5 (0.00% ectopic/artifact intervals)

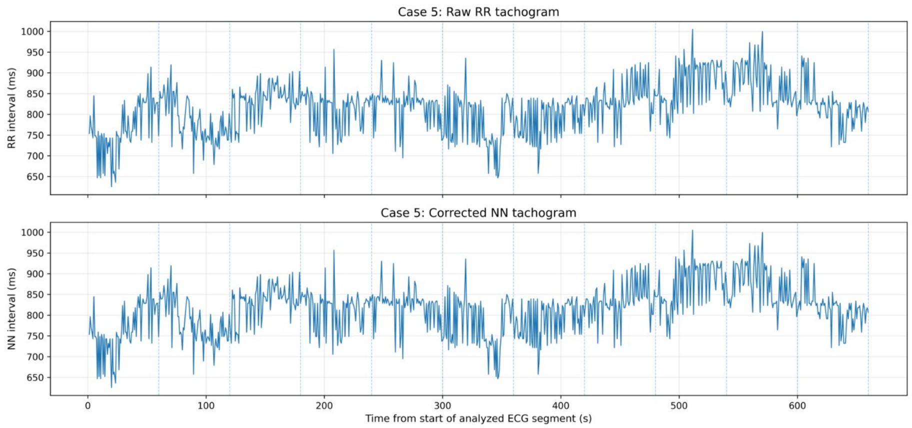

Raw and corrected tachograms for Case 6 (4.09% ectopic/artifact intervals)

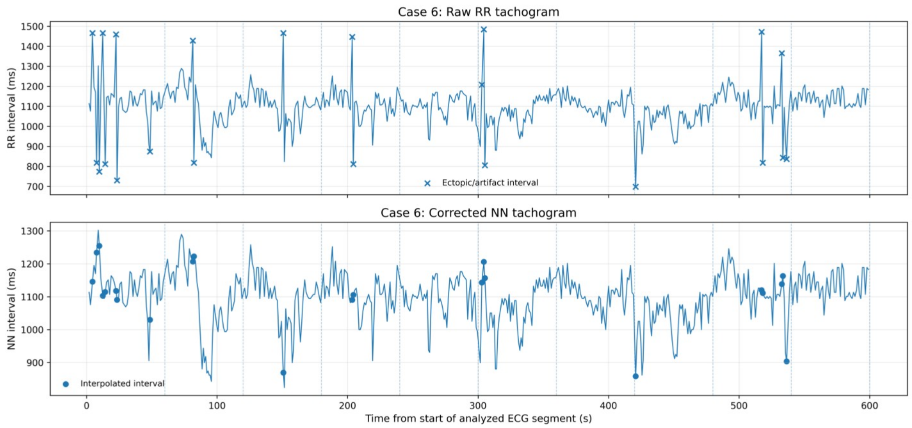

Raw and corrected tachograms for Case 7 (0.61% ectopic/artifact intervals)

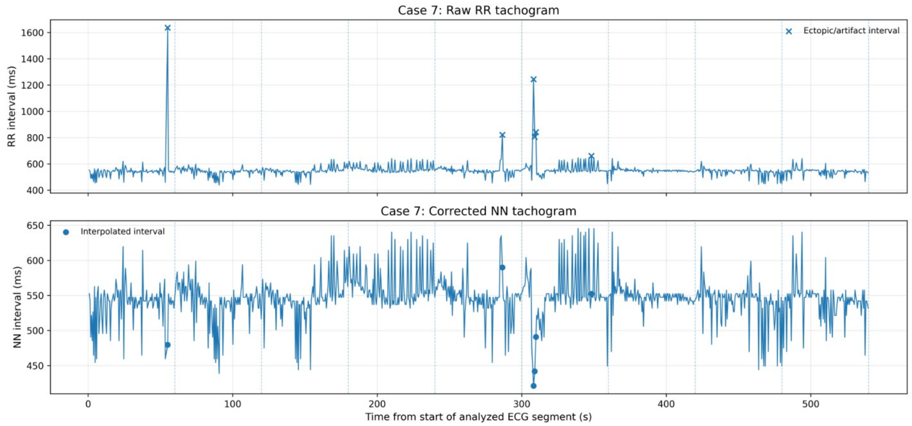

Raw and corrected tachograms for Case 8 (4.78% ectopic/artifact intervals)

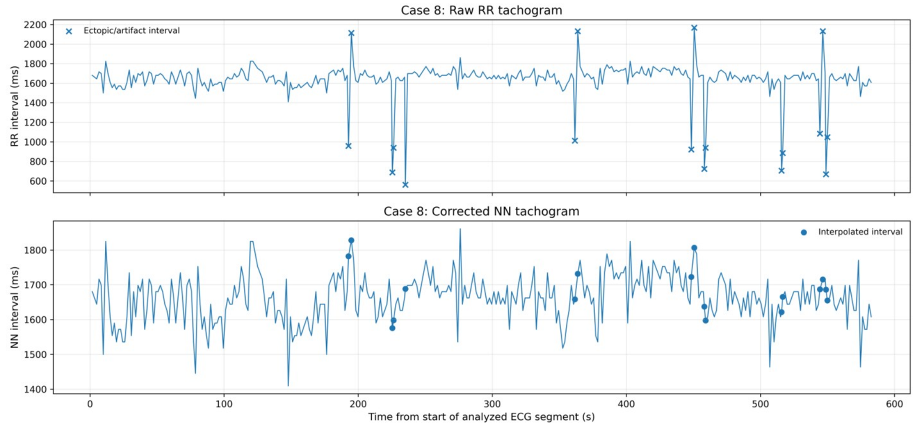

Supplementary Figure S2. Tachogram of the excluded patient with significant atrial dysrhythmia.
The excluded patient showed a high burden of irregular RR intervals, with 49.26% of intervals classified as ectopic/artifact, precluding reliable HRV analysis.

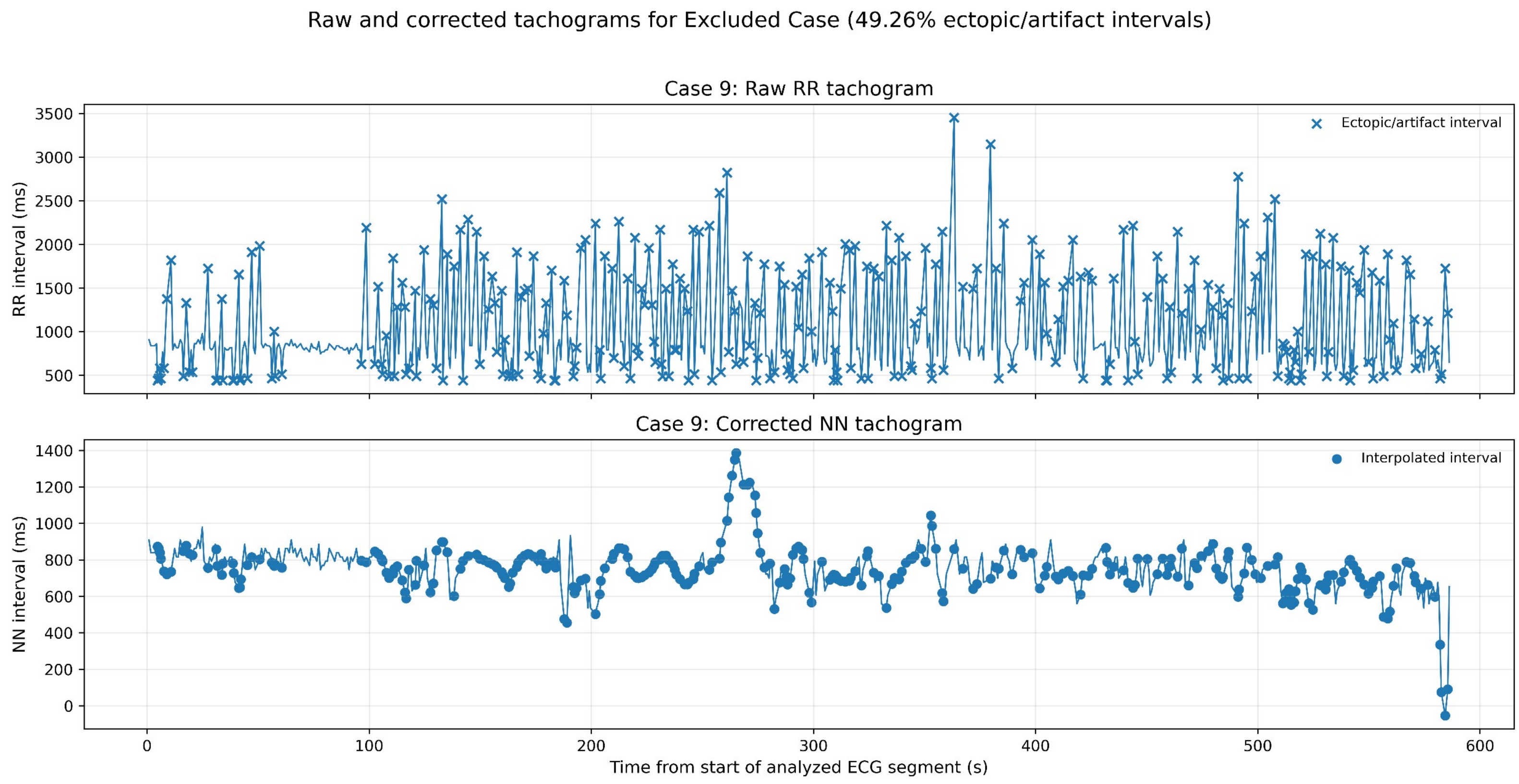

Supplementary Methods S1. Decompression exposure reconstruction and Bühlmann ZHL-16 modeling

Dive profiles were reconstructed retrospectively from the available clinical dive histories and dive-profile information when available. Breathing gas was coded according to the reported exposure: air and compressor-supplied air were modeled using a nitrogen fraction of 0.79, whereas EAN32 exposures were modeled using a nitrogen fraction of 0.68. Gas mixture by case is reported in Table 1.

A custom Python implementation of the Bühlmann ZHL-16 model was used to estimate descriptive decompression exposure variables. Sixteen nitrogen tissue compartments were modeled, with half-times of 2.65, 7.94, 12.2, 18.5, 26.5, 37, 53, 79, 114, 146, 185, 238, 304, 397, 503, and 635 minutes. Ambient pressure was approximated as 1 ATA plus depth/10, and inspired inert gas pressure was calculated according to the nitrogen fraction of the breathing gas.

When repetitive dives were reported, individual dive and surface-interval segments were concatenated into a single time series before tissue saturation was calculated. Therefore, residual tissue inert-gas loading from preceding dives was carried forward into subsequent dives. The maximum gradient fraction was calculated for each tissue compartment across the full reconstructed profile as the ratio between tissue supersaturation above ambient pressure and the corresponding Bühlmann allowable supersaturation. Gradient factors were not applied as decompression-planning modifiers. The reported value represents the maximum fraction of the Bühlmann allowable gradient reached during the reconstructed exposure.

The “compartment of maximum gradient” refers to the Bühlmann tissue half-time compartment, expressed in minutes, in which the highest gradient fraction occurred. Because profiles were reconstructed retrospectively and were not uniformly downloaded from dive computers, these variables were interpreted only as descriptive indicators of decompression exposure and not as formal predictors of decompression sickness severity.
